# Involvement of Autophagy and Oxidative Stress-Mediated DNA Hypomethylation in Transgenerational Nephrotoxicity Induced in Rats by the Mycotoxin Fumonisin B1

**DOI:** 10.3390/toxins15110663

**Published:** 2023-11-17

**Authors:** Nouf Aldawood, Sarah Almustafa, Saleh Alwasel, Waleed Aldahmash, Abir Ben Bacha, Abdullah Alamri, Mohammad Alanazi, Abdel Halim Harrath

**Affiliations:** 1 Department of Biology, College of Science, Princess Nourah bint Abdulrahman University, Riyadh 11671, Saudi Arabia; noaraldawood@pnu.edu.sa; 2 Department of Zoology, College of Sciences, King Saud University, Riyadh 11451, Saudi Arabiasalwasel@ksu.edu.sa (S.A.); waldahmash@ksu.edu.sa (W.A.); 3 Department of Biochemistry, College of Sciences, King Saud University, Riyadh 11671, Saudi Arabia; aalghanouchi@ksu.edu.sa; 4 Genome Research Chair, Department of Biochemistry, College of Sciences, King Saud University, Riyadh 11495, Saudi Arabia; abdullah@ksu.edu.sa (A.A.); msanazi@ksu.edu.sa (M.A.)

**Keywords:** fumonisin B1, kidney, transgenerational effect, oxidative stress, autophagy, DNA methylation

## Abstract

Fumonisin B1 (FB1), a mycotoxin produced by *Fusarium verticillioides*, is one of the most common pollutants in natural foods and agricultural crops. It can cause chronic and severe health issues in humans and animals. The aim of this study was to evaluate the transgenerational effects of FB1 exposure on the structure and function of the kidneys in offspring. Virgin female Wistar rats were randomly divided into three groups: group one (control) received sterile water, and groups two and three were intragastrically administered low (20 mg/kg) and high (50 mg/kg) doses of FB1, respectively, from day 6 of pregnancy until delivery. Our results showed that exposure to either dose of FB1 caused histopathological changes, such as atrophy, hypercellularity, hemorrhage, calcification, and a decrease in the glomerular diameter, in both the first and second generations. The levels of the antioxidant markers glutathione, glutathione S-transferase, and catalase significantly decreased, while malondialdehyde levels increased. Moreover, autophagy was induced, as immunofluorescence analysis revealed that LC-3 protein expression was significantly increased in both generations after exposure to either dose of FB1. However, a significant decrease in methyltransferase (DNMT3) protein expression was observed in the first generation in both treatment groups (20 mg/kg and 50 mg/kg), indicating a decrease in DNA methylation as a result of early-life exposure to FB1. Interestingly, global hypomethylation was also observed in the second generation in both treatment groups despite the fact that the mothers of these rats were not exposed to FB1. Thus, early-life exposure to FB1 induced nephrotoxicity in offspring of the first and second generations. The mechanisms of action underlying this transgenerational effect may include oxidative stress, autophagy, and DNA hypomethylation.

## 1. Introduction

Fumonisins are products of *Fusarium* spp. fungi from which a group of mycotoxins is produced [1]. These mycotoxins accumulate in natural materials as contaminants, most commonly in foods consumed by animals and humans, causing chronic and severe health issues. Fumonisins are divided into four classes (A, B, C, and P); fumonisin B1 (FB1), which is produced by *F. verticillioides*, is considered the most toxic fumonisin and is a common pollutant in agricultural crops such as maize. Progress has been made in developing adequate agricultural approaches to protect against mycotoxin contamination, but some surveys indicate that 70% of raw materials are contaminated by FB1, which means that it is difficult to avoid them since they are produced before, during, and after harvesting. They are sometimes difficult to eliminate, resistant to treatments, and are found in the food of humans and animals. However, FB1 infection can be detected by measuring certain biomarkers, such as the Sa/So ratio in tissues.

FB1 infection commonly occurs in maize-consuming countries, particularly South Africa, Iran, and China. Studies have revealed that South Africa and China have the highest incidences of esophageal cancer due to food contamination with FB1 in humans. Furthermore, because of the climate, improper storage methods, and high levels of FB1 in food, South America, the Far East, and Africa have the highest incidence of FB1 infection [2]. Grains, such as corn and wheat, can be contaminated with these fungi because they are soluble in water [3]. FB1 has been reported to cause liver cancer in rats and other rodents. It can disrupt the intestinal barrier even at low doses and severely harm the duodenum and cecum, but the precise mechanisms underlying these harmful effects are as yet unknown [3]. Additionally, FB1 causes neural tube and neuronal tissue defects in several animals [4], and can interfere with the growth and function of bone in mothers [5]. Furthermore, it has been noted that it significantly increases the weight of the liver while causing a decrease in the overall weight of the animal [6].

Kidney diseases are a significant global health concern and a significant health issue worldwide. It is estimated that nearly 10% of the global population is affected by kidney diseases, and 5–10 million people die annually from kidney disease [7]. The prevalence of kidney disease varies across the globe. Some regions have higher rates due to factors like genetic predisposition, lifestyle factors, unsafe working conditions, and environmental threats. The kidney is widely recognized as one of the organs that is particularly vulnerable to the detrimental impacts of hazardous substances [8]. This susceptibility can be attributed to the fact that approximately 20% of the total cardiac output of blood is delivered to the kidneys where blood undergoes filtration, leading to the concentration of environmental toxins [8]. Consequently, environmental pollutants are known to exert a substantial influence on the development and advancement of kidney diseases. As humans can be exposed to FB1 by consuming contaminated food [2], we aimed in this study to determine the transgenerational effects of FB1 during pregnancy on kidney structure and function in offspring of the first and second generations. We aimed to examine the effects of FB1 on biological processes that are involved in kidney function, such as autophagy, DNA methylation, and changes in oxidative stress factor levels.

## 2. Results

### 2.1. FB1 Induced Histopathological Changes 

**Figure 1:**
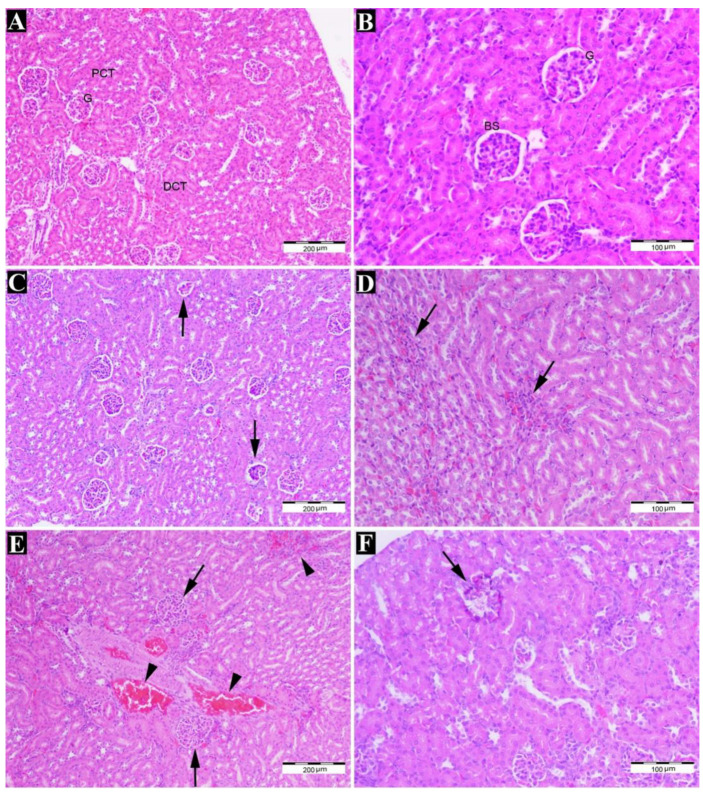
Microphotographs showing histological changes in the kidney structure of treated animals and control animals. (**A**,**B**) Normal structure of the kidney in the control group; the structure and size of the glomeruli (G) and Bowman’s space (BS), the proximal convoluted tubule (PCT), and the distal convoluted tubule (DCT) were normal ((**A**), 100×) ((**B**), 200×). (**C**,**D**) The kidneys of first-generation offspring from the 20 mg/kg and 50 mg/kg treatment groups (**C**) showed glomerular atrophy, expansion of Bowman’s space (arrows), and (**D**) inflammation (arrows) ((**C**), 100×) ((**D**), 200×). (**E**,**F**) The kidneys of second-generation offspring from the 20 mg/kg and 50 mg/kg treatment groups (**E**) showed glomerular hypercellularity (arrows), interstitial hemorrhage (arrowheads), and (**F**) glomeruli calcification (arrows) ((**E**), 100×) ((**F**), 200×) (H&E staining).

The kidney tissue structure was studied by using a light microscope at different magnifications (Figure 1). The results showed histological changes in the kidneys in first-generation offspring rats exposed to 20 mg/kg or 50 mg/kg FB1, such as glomerular atrophy, expansion of the Bowman’s space, and inflammation (Figure 1C,D), compared to the control group. In addition, histopathological examination of the kidneys in second-generation offspring rats exposed to 20 mg/kg or 50 mg/kg FB1 revealed glomerular hypercellularity, interstitial hemorrhage (Figure 1E), and glomerular calcification (Figure 1F) compared to the control group.

### 2.2. FB1 Affected the Number and Diameter of Renal Glomeruli

**Figure 2:**
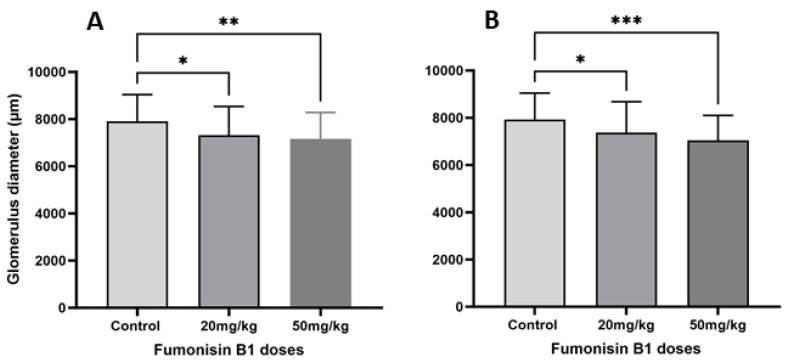
Effect of FB1 on the glomerular diameter in the first (**A**) and the second generations (**B**) in both treatment groups. The results showed that the diameter of the glomerulus was significantly decreased by both doses (20 mg/kg and 50 mg/kg). (*) indicates a *p* value < 0.05, (**) indicates a *p* value < 0.01, and (***) indicates a *p* value ≤ 0.001.

The results revealed that there was a decrease in the diameter of the renal glomeruli in the first generation (Figure 2A) and second generation (Figure 2B) of offspring rats who were exposed to 20 mg/kg or 50 mg/kg FB1 in utero.

### 2.3. FB1 Induced Oxidative Stress in Kidney Cells

**Figure 3:**
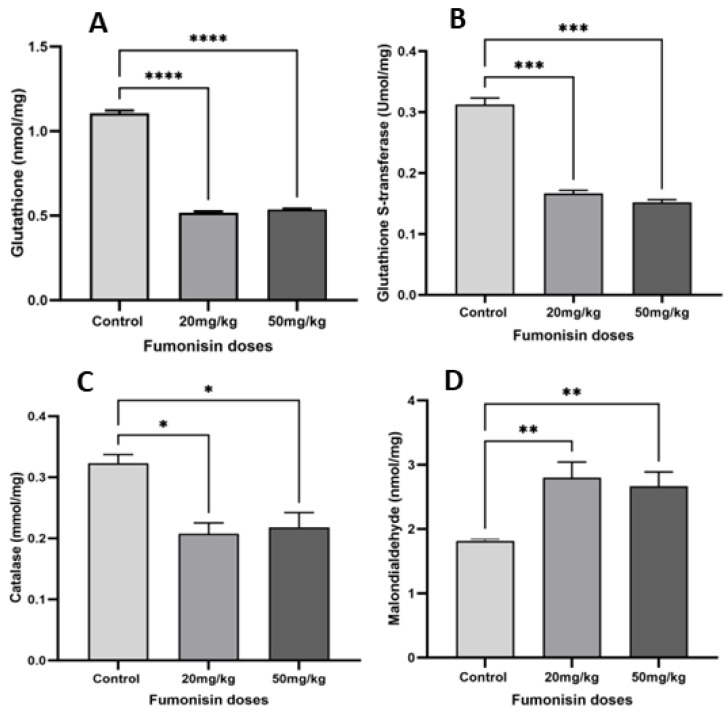
The levels of oxidative stress factors in the kidneys of first-generation offspring in the treatment groups. The results showed a significant decrease in the levels of the antioxidant factors glutathione (**A**), glutathione S-transferase (**B**), and catalase (**C**) with an increase in malondialdehyde levels (**D**) in both treatment groups compared to the control group. (*) indicates a *p* value < 0.05, (**) indicates a *p* value < 0.01, (***) indicates a *p* value ≤ 0.001, and (****) indicates a *p* value ≤ 0.0001.

**Figure 4:**
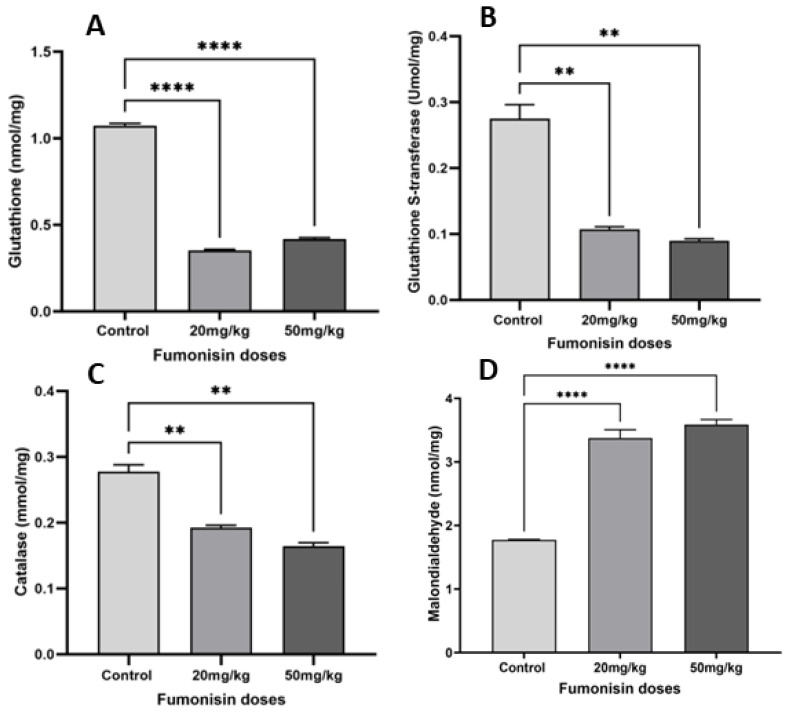
The levels of oxidative stress factors in the kidneys of second-generation offspring in the treatment groups. The results showed a significant decrease in the levels of the antioxidant factors glutathione (**A**), glutathione S-transferase (**B**), and catalase (**C**) with an increase in malondialdehyde levels (**D**) in both treatment groups compared to the control group. (**) indicates a *p* value < 0.01, and (****) indicates a *p* value ≤ 0.0001.

Analysis of oxidative stress revealed a decrease in the levels of antioxidant factors such as glutathione (Figure 3A and Figure 4A), glutathione S-transferase (Figure 3B and Figure 4B), and catalase (Figure 3C and Figure 4C) in both generations of the treatment groups, while malondialdehyde levels significantly increased (Figure 3D and Figure 4D).

### 2.4. FB1 Promoted Autophagy in Kidney Cells

**Figure 5:**
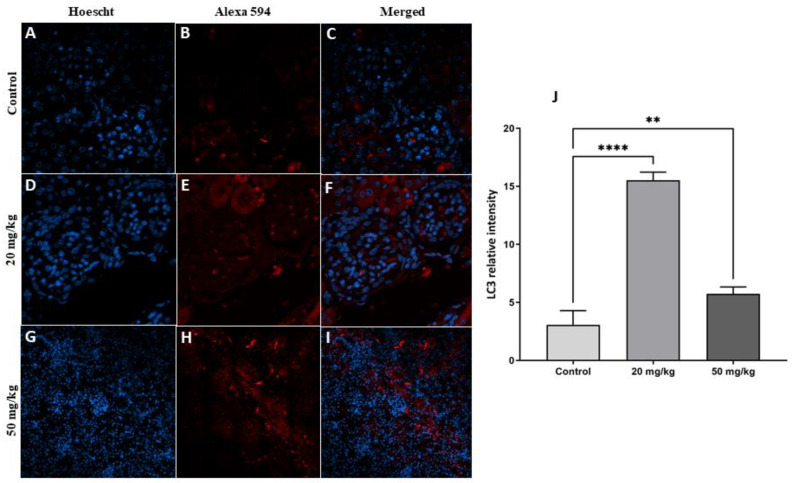
(**A**–**I**) Detection of LC-3 protein with immunofluorescence staining in the kidneys of first-generation offspring rats in the FB1 groups compared to the control group. The nuclei were stained with Hoechst (blue). LC-3 protein was labeled with an Alexa fluorophore (red). (**J**): Quantitative analysis showed a significant increase in LC-3 protein expression in rats that were exposed to 20 or 50 mg/kg FB1 compared to the control rats. (**) indicates a *p* value < 0.01, and (****) indicates a *p* value ≤ 0.0001.

**Figure 6:**
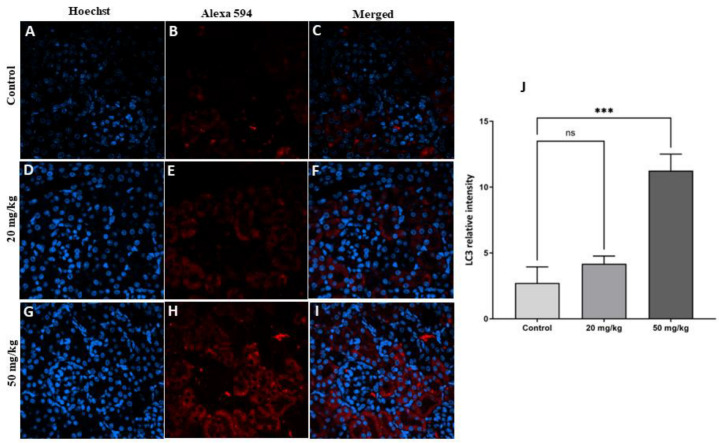
(**A**–**I**): Detection of LC-3 protein with immunofluorescence staining in the kidneys of second-generation offspring rats in the FB1 group compared to the control group. The nuclei were stained with Hoechst (blue). LC-3 protein was labeled with Alexa (red). (**J**): Quantitative analysis showed a significant increase in LC-3 protein expression in rats in both treatment groups compared to the control group. (***) indicates a *p* value ≤ 0.001, and ns: non-significant.

The immunofluorescence technique was used to detect some of the proteins involved in autophagy. The results showed a significant increase in LC3 protein levels in both treatment groups (20 mg/kg and 50 mg/kg) compared to the control group (Figure 5). While the increase was not statistically significant in the second-generation offspring of the 20 mg/kg treatment group, FB1 induced a significant increase in LC3 protein levels in the 50 mg/kg treatment group compared to the control group (Figure 6).

### 2.5. FB1 Induced Hypomethylation in Kidney Cells

**Figure 7:**
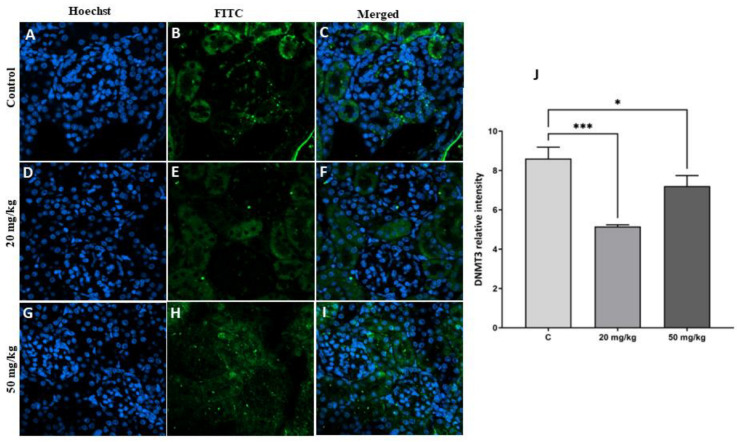
(**A**–**I**) Detection of DNMT3 protein with immunofluorescence staining in the kidneys of first-generation offspring rats in the FB1 groups compared to the control group. The nuclei were stained with Hoechst (blue). DNMT3 protein was labeled with FITC (green). (**J**): Quantitative analysis of the results showed that there was a significant decrease in DNMT3 protein expression in both treatment groups (20 and 50 mg/kg FB1) compared to the control. (*) indicates a *p* value < 0.05, and (***) indicates a *p* value ≤ 0.001.

**Figure 8:**
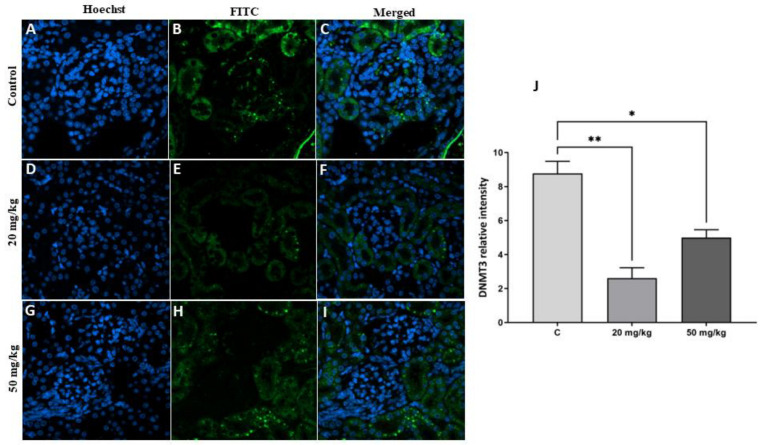
(**A**–**I**) Detection of DNMT3 protein with immunofluorescence staining in the kidneys of second-generation offspring rats in the FB1 groups compared to the control group. The nuclei were stained with Hoechst (blue). DNMT3 protein was labeled with FITC (green). (**J**): Quantitative analysis of the results showed that there was a significant decrease in DNMT3 protein expression in both treatment groups (20 and 50 mg/kg FB1) compared to the control group. (*) indicates a *p* value < 0.05, and (**) indicates a *p* value < 0.01.

The results showed a significant decrease in the level of the epigenetic protein marker DNMT3 in the first-generation offspring of both treatment groups (20 mg/kg or 50 mg/kg) compared to the control group (Figure 7). Similarly, there was a significant decrease in DNMT3 protein expression in the second-generation offspring of the 20 mg/kg and 50 mg/kg treatment groups (Figure 8). The decrease in DNMT3 levels in the second-generation offspring of the treatment groups was more marked than that in the first-generation offspring of the treatment groups.

## 3. Discussion

The kidney is a vital organ that plays an important role in hemostasis and maintaining health, and any injury or damage to the kidney may affect its functions [9]. The kidney is commonly affected by exposure to toxic environmental substances and agents [10]. Many types of environmental pollutants have been linked to the occurrence of acute renal damage following exposure, including fungi and their products [11,12]. However, the toxic effect of the mycotoxin FB1 on the kidneys of offspring and the underlying mechanisms remain unclear.

The results of this study showed that exposure to FB1 caused many histological changes in the kidneys of first-generation offspring, such as glomerular atrophy, expansion of the Bowman’s space, and inflammation. This is in agreement with a previous study showing the occurrence of similar histopathological changes in the renal tubules and the development of fibrosis [13,14]. Moreover, early alterations brought on by FB1 include increased excretion of high- and low-molecular-weight proteins, reduced osmolality, and increased urine volume [15]. Other studies also found an imbalance in kidney function due to increased levels of creatine and urea nitrogen in plasma [15,16,17,18,19,20] and an increase in the excretion of potassium in the urine [21].

Autophagy is a biological process that occurs in eukaryotic cells and is highly dynamic and strictly regulated [22,23]. Autophagy involves the initiation and assembly of the autophagosome, a crucial step regulated by a set of autophagy-related genes and proteins, and the most important protein in this process is LC3. The results of the current study showed that autophagy was triggered in kidney tissue by FB1 exposure because we found an increase in the level of the autophagic protein marker LC3 in the first and second generations of the treatment groups (20 and 50 mg/kg). It has been reported that autophagy is a dichotomous process that initially promotes cell survival but later induces cell death, depending on the context and cellular conditions [24]. Due to the fact that we found many histopathological alterations and a significant decrease in the glomerulus diameter, we hypothesize that FB1 causes nephrotoxicity and that its toxic effect may be related to the induction of excessive autophagy. Several previous studies have shown that autophagy participates in the pathogenesis of nephrotoxicity, leading to apoptotic death of kidney cells [25,26]. For example, as demonstrated by Lim et al. (2012), the administration of cyclosporine A to mice resulted in renal damage and excessive autophagosome production that was accompanied by the expression of active caspase-3 [26]. However, autophagy impairment has been observed to result in kidney damage, indicating the significant protective function that autophagy plays in kidney disease [27]. Previous research has demonstrated that the induction of lysosomal dysfunction by cadmium is achieved through the inhibition of autophagy in acute kidney injury (AKI).

Additionally, FB1 exposure induced oxidative stress in kidney cells in the first and second generations of the treatment groups (20 and 50 mg/kg) as the result of an imbalance between reactive oxygen species formation (increase in MDA levels) and antioxidation, as revealed by the decreases in GSH, GST, and catalase levels. Previous studies have demonstrated that many toxicants can cause kidney tissue damage through oxidative stress, indicating that environmental contaminants have a negative impact on the homeostasis of kidney cells [28,29]. In fact, ROS production resulting from mitochondrial structural disorder and redox homeostasis imbalance leads to cytotoxicity and cell death [30]. As a response to excessive ROS accumulation and oxidative stress induction, excessive autophagy was promoted in the 20 and 50 mg/kg treatment groups, leading to nephrotoxicity, which is considered to be oxidative damage-induced self-destruction. The findings presented in this study are consistent with previous reports establishing the impact of autophagy on cellular function [23,31]. Consequently, we hypothesized that FB1 induces nephrotoxicity and may exhibit a positive association with oxidative stress and increased autophagy in renal cells.

It is now becoming very evident that early-life conditions exert long-term effects on health outcomes during adulthood and that these effects can be transmitted to subsequent generations [32,33,34]. Epigenetics has been identified as a mechanism that is associated with these enduring effects across generations. In particular, DNA methylation is the most comprehensively studied and well-understood epigenetic mechanism. This process typically entails the attachment of a methyl group to CpG dinucleotides located in the regulatory promoter regions of genes [35]. Consequently, this modification, which is regulated by a group of enzymes, including DNA methyltransferases (DNMTs) and methylcytosine binding domain (MBD) proteins, has an influence on the expression of genes [36,37]. There is a growing body of experimental evidence suggesting that alterations in DNA methylation in response to environmental stresses have significant implications in the field of toxicology [38,39]. As an example, alteration of DNMT activity has been observed in mice upon exposure to the pesticide methoxychlor [39]. Our results revealed that the levels of global DNA methylation decreased in the first-generation offspring of both treatment groups, suggesting that DNA methylation levels might be affected by FB1 exposure. These results are in agreement with prior studies showing that FB1 increases DNA hypomethylation in kidney cells [40]; however, Demirel et al., 2015 did not observe modulation of global DNA methylation by FB1 [41]. Furthermore, previous studies have reported the involvement of DNMT3 in the epigenetic suppression of genes for epithelial maintenance of kidney cells, resulting in epithelial–mesenchymal transition that ultimately causes fibrogenesis in rat kidneys [42].

The second-generation offspring did not directly experience the effect of FB1, but their parents directly experienced this effect in utero, which may have directly impacted the development of their kidneys. We found that the levels of global DNA methylation decreased in the second generation in both treatment groups compared to the control. Thus, early FB1 exposure led to persistent epigenetic modification in the kidney cells of the second-generation offspring of the treatment groups despite the fact that their parents did not consume FB1-infected food. Several previous studies have demonstrated that early-life conditions have the potential to impact the methylation of several candidate genes in brain areas that play a crucial role in stress transmission [43]. In fact, during germinal cell formation and embryonic development, epigenetic imprinting and cell differentiation are very active, and DNA methylation contributes to reprogramming cells for future physiological functions [44]. However, the process of reprogramming might result in negative health consequences, such as the development of metabolic syndrome, because epigenetic mechanisms have the capacity to induce phenotypic alterations in response to minimal levels of environmental stressors [45].

## 4. Conclusions

FB1 is a mycotoxin produced by *F. verticillioides* that can contaminate human or animal food, leading to health issues. We found that early life exposure to FB1 causes many histopathological damages to kidney tissues and cells of offspring. Importantly, oxidative stress is an important outcome of FB1 exposure, as indicated by a homeostatic imbalance represented by decreased levels of antioxidant markers (glutathione, glutathione S-transferase and catalase) and increased levels of malondialdehyde. Moreover, FB1 treatment induced excessive autophagy, thereby promoting the development of nephrotoxicity. Interestingly, DNA methylation may play a role in nephrotoxicity induced by FB1. As our current findings do not address the specific DNA methylation changes that occur due to FB1 exposure, further studies assessing alterations in the transcription and methylation of other genes are needed, with the aim of improving our understanding of the particular alterations in DNA methylation that may lead to nephrotoxicity. This research paper presents the findings of a study aimed at understanding the transgenerational effects of the mycotoxin FB1 on kidney structure and function in offspring. The study found that both low (20 mg/kg) and high (50 mg/kg) doses of FB1 caused significant histopathological changes in the kidneys of both first and second-generation offspring. These changes include atrophy, hypercellularity, hemorrhage, calcification, and a decrease in the glomerular diameter. The study also found that FB1 exposure led to a significant decrease in the levels of antioxidant markers such as glutathione, glutathione S-transferase, and catalase, while malondialdehyde levels increased, indicating oxidative stress. Autophagy was induced, as indicated by increased expression of LC-3 protein. The researchers also observed a decrease in DNA methylation, as indicated by a decrease in the expression of the methyltransferase DNMT3 protein. This effect was seen in both the first and second generations, despite the fact that the mothers of the second generation were not exposed to FB1. Thus, the study concludes that early-life exposure to FB1 can cause significant nephrotoxicity in the offspring of the first and second generations. The underlying mechanisms for these transgenerational effects may include oxidative stress, autophagy, and DNA hypomethylation. This research highlights the potential health risk of FB1 contamination in foods and the need for additional research and protective measures to prevent and mitigate exposure to this mycotoxin.

## 5. Materials and Methods

### 5.1. Ethical Approval

This study was approved by the Ethical Committee for the Care and Use of Laboratory Animals at the University of Gafsa, Tunisia (Reference No.: FSG-EA-03-22) and carried out in accordance with the approved guidelines. All experimental procedures are reported in accordance and compliance with the ARRIVE guidelines.

### 5.2. Study Design

Thirteen female and thirteen male rats were obtained from the animal facility of the Zoology Department, King Saud University. Their weights ranged from 200 to 250 g. The rats were allowed to acclimate to a room with a temperature of 22–24 degrees and a dark (12 h)/light (12 h) cycle for one week. They were given free access to tap water and were fed a standard rodent diet *ad libitum*. After mating, the pregnant females were separated into three groups (10 pregnant rats in each group), and on day 6 of gestation, the pregnant females were given the appropriate treatment until delivery. The pregnant female rats in group one received distilled water only and were considered the control group. The pregnant female rats in group two were gavaged with 20 mg/kg b.w./day FB1, while the pregnant female rats in group three were gavaged with 50 mg/kg FB1. The first-generation offspring were obtained after birth and kept until the 28th day of age. Some of them were dissected to collect kidneys, while others were allowed to reach sexual maturity. After mating, pregnant females of the first generation were given free access to tap water and a standard rodent diet *ad libitum* until delivery of second-generation offspring. After 28 days, the female rats of the second generation were dissected, and their kidneys were harvested.

### 5.3. Histopathological Study

The harvested kidneys were fixed in 10% neutral buffered formalin and then dehydrated. Afterward, the tissue samples were incubated with a wax solvent. A Leica microtome was used to cut the wax tissue block into 5 μm sections, the tissue sections were collected on slides. The tissue sections were deparaffinized and stained with hematoxylin and eosin (H&E). Finally, DPX was placed on the samples, and a coverslip was used to cover the samples. The tissue samples were evaluated using a light microscope (Microscope Nikon Eclipse Ni-4). Additionally, the NIS Elements program (version 2.34) was used to measure the diameter of the kidney glomeruli.

### 5.4. Immunofluorescence Staining

Previously prepared slides containing 3 µm thick sections were first placed on a hotplate at 60 °C. The slides were then incubated (two times) in xylene for 10 min to remove the paraffin, and then the sections were exposed to descending ethanol alcohol concentrations (100% twice, 95% twice, 80%, 70%, 50%) for 7 min each for rehydration. The samples were quickly rinsed with distilled water and then placed in phosphate-buffered saline (PBS) for 10 min (3 times). The sections were dried and then exposed to a permeabilization solution (0.2 g sodium citrate, 200 mL distilled water, and 200 L Triton) for 5 min. The slides were immersed in citrate buffer solution (pH 6) and placed in a microwave for 30 s at mid–high level (750 W). The sections were washed three times in PBS for 15 min on a shaker. The sections were blocked with 10% fetal bovine serum (FBS) for 45 min. Afterward, the sections were incubated with anti-LC-3 (1–300 dilution) and anti-DNMT3 (1–1000 dilution) primary antibodies and kept at 4 °C in the dark overnight. The sections were washed three times with PBS for 7 min. Then, the tissue sections were incubated for 45 min at room temperature in the dark with FITC-conjugated anti-rabbit secondary antibody (1:2000 dilutions) (ab6717, Abcam, Cambridge, UK) to detect the anti-DNMT3 antibody, or with Alexa 594-conjugated anti-rabbit secondary antibody (1:2000 dilutions) to detect the anti-LC-3 antibody. Afterward, the tissue sections were washed twice with PBS for 10 min on a shaker and then incubated with Hoechst solution (diluted 1:15,000, Hoechst 33342, Life Technologies, Grand Island, NJ, USA) for 10 min. The tissue sections were washed (3 times) with TE buffer (50 mM Tris base + 1 mM EDTA + 0.5% Triton-x100 in 1000 mL of D.W.; pH 8) for 10 min. Finally, a drop of glycerol and TE buffer (1:1) was applied to the tissue sections, which were then covered with a coverslip, placed in a slide foil, and kept at 4 °C. Using a spinning-disk confocal microscope from Zeiss, DNMT3 and LC-3 signals and nuclear staining were photographed (Germany). Zen 3.1 software was used to examine the immunofluorescence signals of the proteins (ZEN lite, blue edition, Germany).

### 5.5. Oxidative Stress Measurement

Three kidney tissues per group were homogenized in precooled physiological saline using a glass homogenizer. The tissue supernatant was obtained by centrifuging the prepared homogenized solution at 13,000× *g* (4 °C) for 15 min after the cells were lysed by ultrasonication. The concentrations of glutathione (GSH), glutathione S-transferase (GST), catalase, and malondialdehyde (MDA) were assessed in the supernatant. As described in [46], the level of GSH in kidney tissue homogenates was estimated. A total of 1.5 supernatant was added to 1.5 mL of Tris-HCl buffer (200 mM), 0.5 mL of EDTA (pH 7.5) (0.2 mM), 0.1 mL of DTNB (10 mM), and 0.79 mL of methanol. The mixture was vortexed and then incubated at 37 °C for 30 min. To quantify the absorbance at 412 nm, a spectrophotometer was used (Shimadzu (Kyoto, Japan), UV-1800). The level of GST in the kidney tissue was assessed as described in [47]. Catalase (CAT) activity was assessed as described in [48]. Briefly, 20 mL of kidney tissue homogenate was mixed with 1 mL of assay solution, which consisted of 50 mM potassium phosphate buffer (pH of 7.0) and 100 mM H_2_O_2_. At 240 nm, the optical density was determined after 120 s. As described by Buege and Aust [48], malondialdehyde (MDA) levels were determined based on the thiobarbituric acid reaction using a spectrophotometer at an absorbance of 532 nm (Shimadzu UV-1800, Shimadzu, Canby, OR, USA).

### 5.6. Statistical Analysis

The data were subjected to analysis using GraphPad Prism version 9. Statistical comparisons were conducted using one-way analysis of variance followed by Tukey’s multiple comparison test. The level of significance was set at 0.05.

## Data Availability

The data that support the findings of this study are available from the corresponding author (Abdel Halim Harrath) upon reasonable request.
